# Boosting Ultra-Fast Charge Battery Performance: Filling Porous nanoLi_4_Ti_5_O_12_ Particles with 3D Network of N-doped Carbons

**DOI:** 10.1038/s41598-019-53195-1

**Published:** 2019-11-14

**Authors:** Jean-Christophe Daigle, Yuichiro Asakawa, Mélanie Beaupré, Vincent Gariépy, René Vieillette, Dharminder Laul, Michel Trudeau, Karim Zaghib

**Affiliations:** 1 Center of Excellence in Transportation Electrification and Energy Storage (CETEES), Hydro-Québec, 1806, Lionel-Boulet Blvd., Varennes, Quebec J3X 1S1 Canada; 2 Murata Corporation, 10-1 Higashikotari 1-chrome, Nagaokakyo-shi, Kyoto 617-8555 Japan

**Keywords:** Batteries, Organic-inorganic nanostructures

## Abstract

Lithium titanium oxide (Li_4_Ti_5_O_12_)-based cells are a promising technology for ultra-fast charge-discharge and long life-cycle batteries. However, the surface reactivity of Li_4_Ti_5_O_12_ and lack of electronic conductivity still remains problematic. One of the approaches toward mitigating these problems is the use of carbon-coated particles. In this study, we report the development of an economical, eco-friendly, and scalable method of making a homogenous 3D network coating of N-doped carbons. Our method makes it possible, for the first time, to fill the pores of secondary particles with carbons; we reveal that it is possible to cover each primary nanoparticle. This unique approach permits the creation of lithium-ion batteries with outstanding performances during ultra-fast charging (4C and 10C), and demonstrates an excellent ability to inhibit the degradation of cells over time at 1C and 45 °C. Furthermore, using this method, we can eliminate the addition of conductive carbons during electrode preparation, and significantly increase the energy density (by weight) of the anode.

## Introduction

Despite the existence of widespread environmental regulations, pollution and global warming still present a tremendous challenge for humanity^1,2^. Electrical vehicles (EV) and energy storage (ES) devices are gaining popularity for daily use^3^. However, since challenges persist, the scientific community must develop safe, long-lived, and fast charge-discharge batteries in order to promote the use of such vehicles and ES^4,5^. Our research group^6–12^ as well as scientists^13–15^ around the world believe that LiFePO_4_, lithium iron phosphate (LFP) and Li_4_Ti_5_O_12_, lithium titanium oxide (LTO)-based batteries are ideal candidates to meet these needs for ES applications.

Lithium titanium oxide (LTO) holds promise as anode material for rapid-rate charge-discharge batteries. Carbon coated LTO (LTO-CC) has reportedly been used successfully as anode material in 18,650 cylindrical cells^11,12^. The carbon layer is a good method to form a protective coating, and consequently moderate the degradation of the electrolyte in contact with LTO and residual water (residue from the cathode), thereby inhibiting gas evolution^16,17^. In the case of pouch architecture, the cell tends to expand, which can be a safety risk^18,19^. Moreover, the carbon coating of LTO makes it an electronically conductive and effective battery material^9,11^. Good electronic conductivity is a key factor in achieving an ultra-fast charge-discharge cell with high capacity retention, and decreasing the charge transfer resistance of the electrode^16,20^. Industrially, the carbon coating formed by the pyrolysis of sucrose creates an amorphous but uniform nano-layer of carbons on the surface^21,22^.

Comprehensive contemporary literature exists exploring the applicability of various types of carbon for use in batteries. Active particles wrapped with graphene or graphene-oxide demonstrated promising results as both cathode^23,24^ and anode^25^ materials; for example, sulfur particles wrapped with graphene demonstrated outstanding stability for Li-S batteries^26^. Also, N-doped nano-carbons exhibited great potential for application as materials in batteries because of their higher electronic conductivity^20,27–29^. Basic nitrogen can also interact with titanium to form TiN (Ti^3+^ is more conductive), in the case of titanium-based electrode materials. Furthermore, presence of defects increased the number of active sites, and consequently the Li^+^ permeability through the carbon coating^30^. Anodes based on this material were used in various batteries including lithium-ion^31,32^, lithium-air^27^ and potassium^33^-based units. Anodes manufactured with carbon nanotubes doped with nitrogen showed a discharge capacity 1.5 times greater than those made of un-doped material^29^. Composites of nitrogen-doped carbons with LTO^34^, Sb_2_S_3_^35^, and Sn^36^ were also successfully applied to lithium ion batteries (LIB) with very good electrochemical performance; specifically, higher capacities at high C rates and cycle life were recorded.

In this paper we report the development of a new eco-friendly and scalable method of forming a thin layer of nitrogen-doped carbons on an LTO surface, resulting in successfully filling all the nanopores of the particle with carbons. This technique allows the formation of a uniform 3D pathway of electronic conduction, inside and outside the LTO particle. That is the first example of N-doped carbons filling LTO porous particles. The particles are then protected by a carbon coating that is able to limit the degradation of the battery as well as reduction of the resistance of the electrode. Moreover, the charge capacity at 10C increased by 44%, and the specific energy density of the anode increased by the elimination of superfluous carbons in the electrode preparation.

The most commonly used method to apply carbon coating to inorganic materials such as LFP, LTO, and TiO_2_, which are involved in the preparation of lithium batteries, is to use sugar or its derivatives as a carbon source. The sugar is mixed with the active material and carbonized at high temperature. This technique was introduced by Armand *et al*.^21,22^ for LiFePO_4_, and is routinely used for LTO^37^. It is an inexpensive and scalable method which permits the formation of a nanolayer. Many carbon sources (polymers) were reported; however, a majority of them involve mechanical mixing with inorganic particles^38–41^. Since polymers cannot diffuse inside the pores because of the nature of the chains, no example was found in the literature for a method facilitating this. Toluene was also investigated as a carbon source. The carbon painting was done by vapor deposition, resulting in a uniform carbon coating of the particles with high electronic conductivity^16^. Recently, few publications have reported the use of organic substances^42,43^ or polymers^44^ (folic acid, pyrrole, etc.) as the source for N-doped carbons; however, all are using classical mechanical mixing methods such as ball milling which does not allow the formation of a homogeneous coating. Our study was also motivated by a recent development: Posco stopped the production of carbon-coated LTO and replaced this product with a doped LTO. Therefore, the surface protection of LTO by carbons is no longer available for large-scale battery production. We therefore decided to develop a new, affordable, eco-friendly, and scalable method to produce N-doped carbon-coated LTO.

As reported previously by our research group^6^, commercial LTO is formed by the agglomeration of primary particles of ~10 nm into secondary particles of a few micrometers. This LTO structure i.e. nanoporous micro-spheres, facilitates optimal performance when it is used as an anode material^45,46^. However, another challenge arises from that structure: in order to optimize the coating and the electrochemical performances, we must develop a method to fill every pore of each secondary particle and fully coating every single particle. Such a method was not yet industrially developed.

**Scheme 1:**
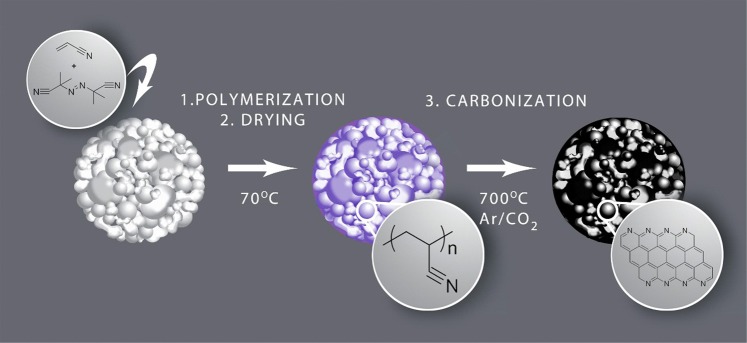
Illustration of the carbon coating LTO process (Scheme was drawn by Ms. Eloïse Leroux).

We selected poly(acrylonitrile) as a carbon and nitrogen source because the processes for producing carbon fibers and porous carbons, as well as the synthesis of low-cost N-doped carbons^47,48^ are well-known. The polymerization of acrylonitrile can be easily accomplished by free radical polymerization. To achieve a greener process, we performed interfacial suspension polymerization in water to absorb the monomer and the radical initiator onto and inside of the inorganic particles using high-energy sonication. This method allows the fluid organic mixture to diffuse inside the pores and fill the LTO secondary particles, resulting in adsorption onto the primary particles. An additional factor that makes the absorption possible is the occurrence of the process in water, due to which, LTO acts as surfactant. Thereafter, the polymerization is conducted for 16 h at 70 °C under vigorous stirring. A uniform nano-layer of carbons on the surface of the micro-particle was achieved by drying the aqueous dispersion using Spray-Dryer. This was used to form a homogeneous carbon-coating on silicon nanoparticles, resulting in tremendous electrochemical performance as anode material^49^. This method had two objectives: removing water, and forming a uniform nano-layer of polymer on the LTO surface. The N-doped carbons are formed by carbonization under CO_2_/argon at 650 °C and 700 °C^45^. Scheme 1 illustrates all processes, and the experimental details are described in the Experimental section.

## Results and Discussion

### Physical properties of the carbon coated LTO particles

Table 1 reports the physical characteristics of the N-doped carbon-coated LTO particles (LTO-CC) selected for this study. Carbonization temperatures, 650 °C and 700 °C, were selected because the sintering of LTO occurs at higher temperatures^50^. As can be seen from Table 1, the temperature affects the conductivity of the carbons (LTO-CC1 vs LTO-CC2). Therefore, we can assume that a part of the aromatic ladder formed during the first step at 240 °C was not completely oxidized into activated carbon. Furthermore, the carbon is amorphous as demonstrated by the D/G ratio obtained by Raman spectroscopy (Fig. S1 in SI); the D band denotes the presence of disordered carbons and the higher ratio between D and G bands is related to the defective (amorphous) carbons^51^. Amorphous carbons are more suitable for battery applications because they enhance the quality of lithium diffusion^16,52^. The existence of N-doped carbons was confirmed by the detection of nitrogen through elemental analysis (Table 1) and EELS analysis for LTO-CC3 (Fig. S3 in SI). Moreover, the FTIR spectrum showed distinctive peaks for C=C, C=N and C-H rising from aromatic structure. A detailed spectrum can be found in Fig. S4 in SI.

**Table 1:** Physical characteristics of carbon-coated LTO particles.

ID	T carbonization (°C)	D/G^a^	%C^b^	%N^b^	Specific Surface^c^ (m^2^ g^−1^)	Conductivity^d^ (nScm^−1^)
LTO-CC1	650	2.5	1.1	0.30	6.7	1.0
LTO-CC2	700	2.9	0.84	0.16	9.5	20
LTO-CC3	700	3.1	0.49	0.14	8.1	4.0
LTO-CC4	700	2.6	0.19	ND	7.1	200

^a^Determined by RAMAN spectroscopy; ^b^Determined by elemental analysis; ^c^Determined by BET; ^d^Measured on compressed powder by 4-probes resistivity meter. ND: not determined, under the limit of detection.

Okada *et al*.^45^ and Ko *et al*.^53^ reported the use of argon/CO_2_ gas during carbonization as a way of creating porous carbons associated with an increasing of specific surfaces evaluated by BET analysis. This procedure was adopted by the need to increase the wettability and lithium diffusion of LTO. Therefore, we observe that the carbon coating increases the specific surface approximately by a factor of two (Table 1), which improves the wettability and lithium diffusion of the active particles^54^. The initial specific surface reported by the supplier is 4 m^2^g^−1^ for the LTO precursor (LTO-reference). The conductivity reported in the Table 1 is measured in terms of the function of the pressure applied to the powder. It should be noted that LTO-CC4 resulted in a higher value due to the presence of carbon chunks not coating on the LTO particles in the sample (visualized by SEM); these chunks increased the conductivity by creating the contact between the particles under pressure, acting as free conductive carbons. The nitrogen content was also not determined because under limit of detection of the apparatus.

**Figure 1:**
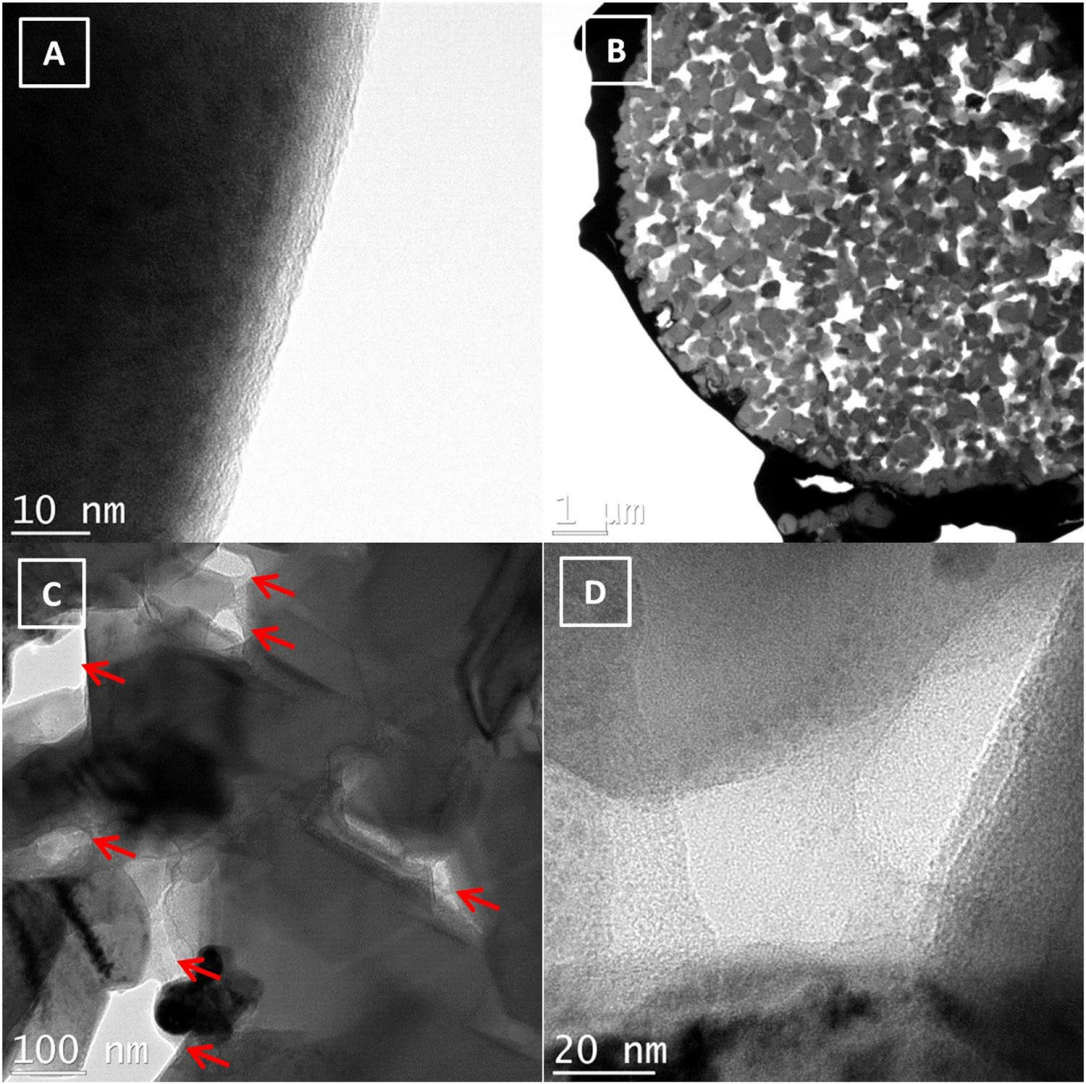
(**A**) Surface of lithium titanium oxide (LTO) with a layer of N-doped carbon. (**B**) Cross-section of carbon-coated lithium titanium oxide (LTO-CC3). (**C**) Magnified images inside the cross-section. Carbon-filled pores are marked by red arrows. (**D**) Magnified images inside a pore filled by N-doped carbons.

**Figure 2:**
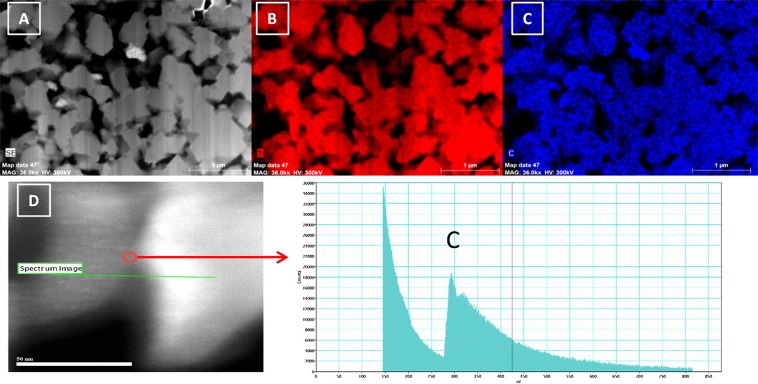
(**A**) Images of particles after fibbing. (**B**) Mapping of titanium. (**C**) Mapping of carbon. (**D**) Images of two primary particles allowing analysis of their junction (scale bar span 50 nm). Electron energy loss spectroscopy (EELS) profile showing the presence of carbons (300 eV and a count of 18000) between the two particles.

The images recorded by high-resolution transmission electron microscopy (TEM; Figs 1 and 2) show the effectiveness of the method for forming a thin and uniform carbon layer on the nanoparticle surfaces. Moreover, focused ion beam (FIB) cross-sections of particles were performed to prove the presence of carbon inside the secondary particles, and to paint primary particles. We expected a covering of the primary particles, a filling of the pores, and presence of carbon junctions between two primary particles. All these features should offer an advantage in the formation of a high performance LTO for battery application because the electronic network would cover all the active particles in 3D linkage. Figure 1 shows images of LTO-CC3 surfaces and cross-sectional images after cryo-fibbing a secondary particle (Fig. 1B). Figure 1A shows a thin layer of N-doped carbon on the LTO surface; this layer was ~5 nm in thickness, which is optimal for lithium diffusion (also see Fig. S3 in SI)^16^. Fig. 1B shows the secondary particle after fibbing. Myriad nanoparticles filled with N-doped carbons can be observed in Fig. 1C,D; many pores are filled (red arrows) and edges of primary particles are coated. Because carbon coating can be found in the center of the secondary particles, we can assume that was not an effect of the fibbing. All the 3D particles can promote the transfer of electrons which favors better electronic contact and thereby makes more active materials accessible for electrochemical reactions. Moreover, the coating is homogeneous and uniform inside and outside the particles as demonstrated in Fig. 2.

Mapping of titanium and carbon inside the secondary particles after fibbing (Fig. 2B,C), demonstrates the perfect coating of every single primary particle achieved with our method. Similar analysis was performed on commercial LTO-carbon coated from Posco (Fig. S4 in SI), and only chunks of carbon, no homogeneous coatings, were detected inside the secondary particle. Furthermore, to demonstrate the effectiveness of our method, Fig. 2D depicts the electron energy loss spectroscopy (EELS) spectra at the junction between two primary particles. Herein, the pores as well as the small spacing between two primary particles were filled with the N-doped carbon coating (see also Fig. S3 in SI). This was possible because the pores were first filled with organic monomers and initiator before the start of the polymerization; this was promoted by the suspension interfacial polymerization and by the sonication. We thus demonstrated that all the particles (primary and secondary) are totally covered by N-doped carbons; we believe this coating contributes significantly toward more efficient electronic and ionic pathways. Recently, folic acid was reported for forming an N-doped carbon by a ball-milling process^43^, however the coating was not homogenous, probably as a consequence of the fabrication method. No previously reported method has been found to fabricate such a 3D N-doped carbon network.

### Electrochemical performances of batteries

One of the major challenges we had to circumvent with our new material was the fabrication of electrodes. As the LTO was completely covered with N-doped carbons, less adhesion occurred on the aluminum collector with PVDF or SBR/CMC when they were used as binders. Bubbles (gas evolution) were formed on the surfaces of the electrodes, and they were not suitable for cell assembly because their surfaces were not exploitable. Wan *et al*. (2012) reported the reaction of graphene oxide with aluminum at low temperatures to form oxygen^55^. Furthermore, N-doped carbon is known to be a good catalyst for oxygen reduction reactions. We believe a similar behavior occurs by reaction of aluminum oxide (layer on aluminum) with basic nitrogen from carbon^56,57^. This effect became significantly important with the decrease of carbon content on LTO, requiring a simple approach to preventing the reaction and optimizing the adhesion: poly(acrylic acid) (PAA) was used as a co-binder with SBR (replacing CMC). As demonstrated previously by Claverie group^58,59^, poly(acrylic acid) can be used to effect a suitable dispersion of TiO_2_; because LTO has a similar structure, we assume it has the same behavior. The negative charges of polymers interact with the surface of the particles (basic nitrogen from carbons) while the hydrophobic polymer backbone is against the current collector. The polymer acts as a dispersant, and builds a “wall” between the current collector and the coating; similar interaction was also reported for PAA with PANI particles^60^. Subsequently, by neutralizing basic nitrogen with the remaining acid groups and by using its amphiphilic property^61^, gas evolution was stopped because a protective film was formed between the aluminum oxide and the active particles (Fig. S5 in SI). That is a probable mechanism based on our observations and review of the literature; we know electrodes fabricated with PVDF and SBR-CMC generated bubbles on their surfaces (gas); using PAA, no such bubbles were observed. Gas generation destroys the surface of the anode and make it less appropriate for use. Moreover, the use of PAA had the additional advantage of increasing the adhesion with aluminum by the presence of numerous polar groups; therefore, two problems were solved with this polymer.

To evaluate the capacity of LTO-CC that prevents degradation in the cell, we performed a float test using half cells (LTO-Li). We reported this test in a previous publication for the evaluation of the materials to prevent degradation by promoting side reactions, and subsequent aging of the cell^6–8^. The electrochemical conditions of the float test are described in the Experimental section. Electrodes with LTO-CC3 and LTO-CC4 were evaluated and compared with an LTO reference and LTO-CC reference. It was found that LTO-CC3 experienced virtually no degradation (capacity retention was 100%) and LTO-CC4 experienced 98.0%, while the LTO reference and LTO-CC reference experienced 94.7% and 97.6% of capacity retention, respectively. Therefore, we can assume our carbon coating is better than those prepared by commercial processes at preventing degradation. Coating of carbons^10^ or polymers^6,7^ on LTO particles was already known to be efficient in mitigating side reactions. It appears that a better covering, as observed in our case, permits the elimination of degradation after the float test. Furthermore, LTO electrodes constructed with PVDF have shown higher degradation than electrodes manufactured with SBR/CMC (85.2% for PVDF) (Fig. S7 in SI).

**Figure 3:**
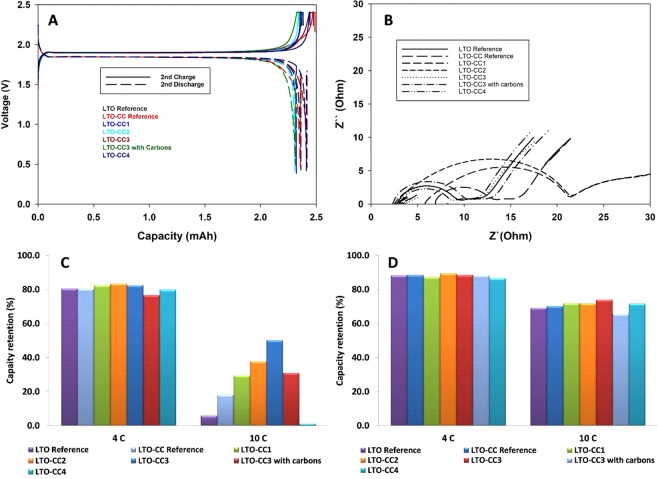
(**A**) Charge-discharge profiles at 0.2C and 25 °C of full cells. (**B**) Nyquist plots of the cells after formation (0.2 C, 25 °C). (**C**) Bar diagrams of capacity retention after fast charge. (**D**) Bar diagrams of capacity retention after fast discharge.

Coin cells with carbon-coated LiFePO_4_ cathodes and N-doped carbon coated LTO anodes were assembled to evaluate the efficiency of our system. LTO references were made with an SBR/CMC binder because we used a water-based binder for our samples, and because Chou *et al*. demonstrated the superiority of using an SBR/CMC binder with LTO for high C rate performance^62^. We used a load mass of 10 mgcm^−2^ for the anode composition, in order to meet the requirements from the industry with regards to high energy density. The loading and the thickness of the electrodes have an important impact on rate performances. The capacity values were recorded at 0.2C and 25 °C for 2 cycles, and varied between 150 and 170 mAhg^−1^. These values were valuable for evaluations, and were used to calculate the retention capacity subsequently reported for the other tests. Figure 3A shows the capacity-voltage profile for the samples and the references. No major changes were observed in the shape of the curves; unsurprisingly, the LTO-reference without carbon coating demonstrated a higher capacity as more active materials were used in the composition of the electrode. We believe there was no contributions of the N-doped carbons on the capacities recorded because the amount were not significant (~1 wt.%), and the increasing of nitrogen contents in carbons were not related with better electrochemical performances (LTO-CC1 vs. LTO-CC3) as reported by Y. Li and Q.-H. Wu in 2017 for nitrogen-doped carbon nanotubes used as anode materials^31^.

Furthermore, no additional carbons (Carbon black, Carbon fibers, etc.) were used as conductive materials in the fabrication of the electrodes; these were composed of 96 wt% LTO-CC, whereas the references were composed of 90 wt% LTO. We found that no extra carbons were required to achieve performance similar or superior to those achieved by state-of-art electrode composition. This is a major advantage for industry because it allows the increase of energy by weight for an anode. This advantage is particularly evident when we consider Fig. 3B,C. The initial capacity of LTO-CC3 with carbons is lower when the electrode is created without carbons (LTO-CC3) (Fig. 3A), and the resistance recorded for LTO-CC3 was slightly lower comparing with the sample with carbons (Fig. 3B). Moreover, in Fig. 3C,D all the charge-discharge retention capacities at 4 C and 10 C are higher for the sample without carbons. We believe the quality and the perfect covering of all the primary particles with our N-doped carbons dramatically enhances the performance at high C rates and eliminates the use of conductive carbons; bad coverings like LTO-CC4 (presence of carbon chunks not coating on particles) are disadvantageous as shown in Fig. 3DC. All other samples (LTO-CC) demonstrated superior retention capacities at high C rates compared with references. LTO-CC3 had a retention capacity of 50.2% after charge at 10C, whereas the LTO reference and LTO-CC reference had retention capacities of 5.8% and 18.2%, respectively a significant performance increase. LTO-CC reference only has chunks of carbons (commercial product) as observed by HRTEM (Fig. S2 in SI), therefore not all the particles were coated and could contribute to the reaction with lithium because of this lack of electronic conductivity; we believe this coating is not optimized for high rate performances. Our method allows the coating of every single particle (Figs 1 and 2), creating a perfect pathway for electron diffusion, and subsequently maximizing the contribution to the electrochemical reaction of the LTO. The optimization is reflected by the high retention capacity at 10C after the charging of the cell with LTO-CC3.

Here, we demonstrated that good electronically conductive materials have a major impact on fast charge performance while insulating material such as poly(styrene) impedes the diffusion of electrons and lithium despite providing good protection against degradation^6^.

**Figure 4:**
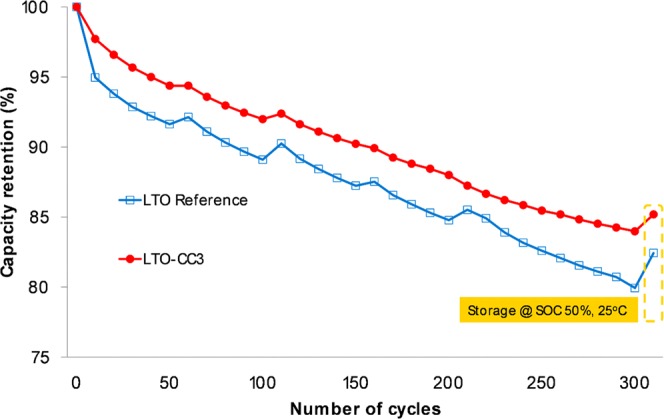
Cycle-life performances of LFP-LTO cells (1C, 45 °C).

Because a long cycle-life cell is an important feature of an LFP-LTO system, the evaluation of cycle life is critical to validating the efficacy of our method. Coin cells composed of LTO-CC3 (anode) and carbon coated LFP (cathode) were assembled and cycled at 45 °C and 1C for 300 cycles. Because of the use of water-based binder, the sample was compared to an anode made with LTO and SBR/CMC binder. Figure 4 shows the retention capacity as a function of the number of cycles. Available commercial products, i.e., LTO without a carbon coated layer, were also tested for comparison to have a better insight into the performance a potential commercial battery. As can be seen in Fig. 4, the most significant degradation occurs after the first ten cycles. This effect is more significant in the case of bare LTO, as we reported in a previous publication^6^, and therefore it is clear that LTO-CC effectively prevented degradation at the beginning of the cycles, and retained this stability for the rest of the test. Capacities were recorded at 0.2C and 25 °C every 50 cycles; it can be seen that the reference was more affected by the intensive cycling at 45 °C. Moreover, after 300 cycles, LTO-CC3 showed a higher retention capacity by 3.5%. The carbon layer acted effectively to protect against degradation like a polymer coating, and therefore we believe that in this case carbon only plays the role of a protective layer. The good covering of primary particles (LTO) was also advantageous in producing more durable cells.

## Conclusion

In conclusion, we present a new method of forming a carbon coating on and inside LTO particles. We believe that it is a promising and eco-friendly approach toward optimizing the coating of LTO particles on a large scale. Polymerization of acrylonitrile by interfacial dispersion polymerization allows a perfect 3D coating of LTO with N-doped carbons. The use of porous and active carbon plays a major role, in conjunction with this method, toward improving battery performance. For the first time, TEM images demonstrated an unequivocal coating inside the pores and in the junction between primary particles. This unique carbon painting facilitates an impressive enhancement in the high C charging-rate and cycle life. Furthermore, we increased the energy by weight of the anode by eliminating superfluous conductive carbons in the electrode preparation. In summary, we demonstrated the effectiveness of using our method to create a 3D network of N-doped carbons, with every single nanoparticle perfectly coated in order to be active as electrochemical material. As such, we are confident that this new method can overcome the current technological challenges related to LTO-based batteries and transcend the targets for battery performance.

## Methods

### Experimental section

Acrylonitrile was passed over a bed of basic Al_2_O_3_. Azobisisobutyronitrile (AIBN) was purified by recrystallisation in methanol and dried under vacuum for 12 hours. The monomers were used immediately after purification. The carbon coated lithium iron phosphate (LFP) was purchased from Sumitomo Osaka Cement and the lithium titanium oxide (LTO) from Posco. PVDF, LIPF_6_, and the carbonate solvents were obtained from BASF. The carbon Denka Black was from Denka. The SBR latex and CMC were obtained from Zeon Co. and DKS Co. respectively. All other chemicals from Sigma Aldrich and Acros were used as received. Water had a resistance of 18.2 MΩcm^−1^. Same materials were reported as a state-of-art materials in our previous publication cited in refs.^6,7^.

#### Polymerization of acrylonitrile in presence of LTO (LTO-CC3)

LTO (60.0 g) was dispersed in water (300 ml) by sonication (3 min. at 70%) and stirring. Acrylonitrile (9.0 g, 170 mmol) with AIBN (90.0 mg, 0.55 mmol), previously dissolved in monomer, was added in the dispersion under stirring. Sonication was applied for another 3 min. The solution was stirred for 30 min. under a flow of nitrogen. The flask was connected to a condenser. The solution was heated at 70 °C with vigorous stirring (750–1000 rpm) under nitrogen. After 16 hours, the dispersion was cooled, and subsequently dried by Mini Spray Dryer B-290 from Buchi, the sample was heated at 180 °C, the pump being used at 25% and the blower at 95–100%. An off-white powder was recovered. The carbonization of the powder was achieved in a tube furnace (MTI OTF-1200X). The ramp was 25 °C to 240 °C at 5 °C min^−1^ under air. We kept the sample at this temperature for 1 hour. Thereafter, the temperature was raised to 700 °C at a rate of 5 °C min^−1^ under argon/CO_2_.

#### Experimental characterization

The FIB sample was prepared with a NB5000 from Hitachi using gallium atoms. The sample was cooled to ~−100 °C to minimize damage to the C layer through the FIB preparation. The TEM images were obtained with a High-Resolution environmental TEM (Hitachi HF3300). EELS data were obtained in STEM mode using a Quantum ER GIF system from Gatan. Raman spectra were recorded with a LabRam Aramis from Horiba. Determinations of carbon and nitrogen contents were obtained by elemental analysis by using CS230 from Leco. BET measurements were taken using a QuadraSorb Station 3 from QuantaChrome. Electronic conductivities were measured with a 4-probes resistivity meter on compressed powder under various pressures with a Hiresta GP Module (PD-51 model) resistivity meter from Mitsubishi Chemical Analytech. FTIR measurements were conducted on the CARY 630 from Agilent.

### Cells assembly and electrochemical measurements

LFP-LTO cells were assembled and tested as described in our articles^6,7^; for the fabrication of anodes, this procedure was used for those containing Li_4_Ti_5_O_12_ (LTO) or LTO-CC, carbon black, and SBR/CMC or SBR/PAA as binder in a proportion of 91:5:2.5/1.5, respectively. PAA had a molecular weight of 450 000 gmol^−1^. The slurry was coated on a 15 μm aluminum collector, using the doctor blade method. When no conductive carbons were used, a proportion of 96:2.5/1.5 was applicable.

## Supplementary information

Supplementary information
