# Supplementary material for: Boosting Ultra-Fast Charge Battery Performance: Filling Porous nanoLi4Ti5O12 Particles with 3D Network of N-doped Carbons
Source: Sci Rep. 2019 Nov 14;9:16871. doi: 10.1038/s41598-019-53195-1 (PMC6856524; doi:10.1038/s41598-019-53195-1)
Supplement: Supplementary file 1 — Supplementary information [file 41598_2019_53195_MOESM1_ESM.pdf]

# Supporting Information

## Boosting Ultra-Fast Charge Battery Performance: Filling Porous nano$Li_4Ti_5O_{12}$ Particles with 3D Network of N-doped Carbons

Jean-Christophe Daigle[1], Yuichiro Asakawa[2], Mélanie Beaupré[1], Vincent Gariépy[1], René Vieillette[1], Dharminder Laul[1], Michel Trudeau[1], and Karim Zaghib[1*]

[1]Center of Excellence in Transportation Electrification and Energy Storage (CETEES), Hydro-Québec, 1806, Lionel-Boulet Blvd., Varennes, Quebec J3X 1S1, Canada

[2]Murata Corporation, 10-1 Higashikotari 1-chrome, Nagaokakyo-shi, Kyoto 617-8555, Japan

## Figures

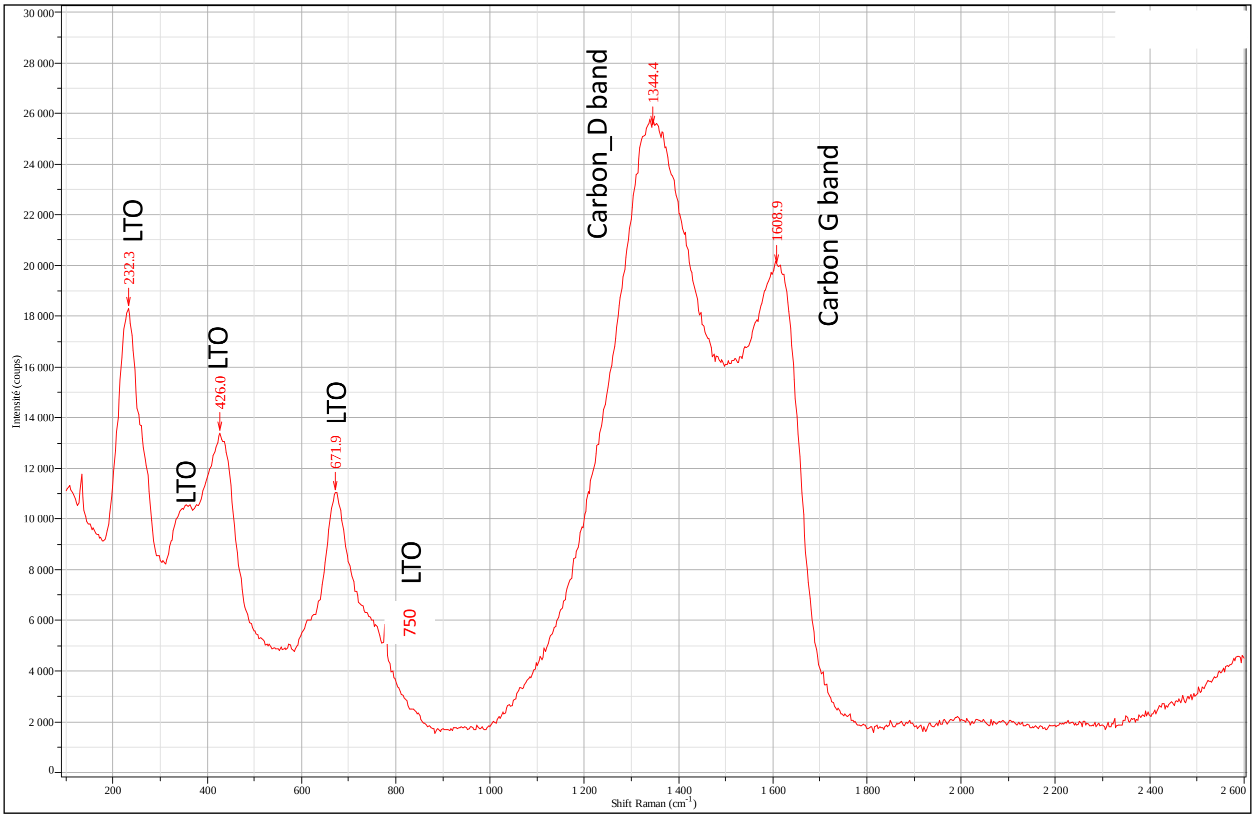


**Figure S1.** RAMAN spectrum of LTO-CC2

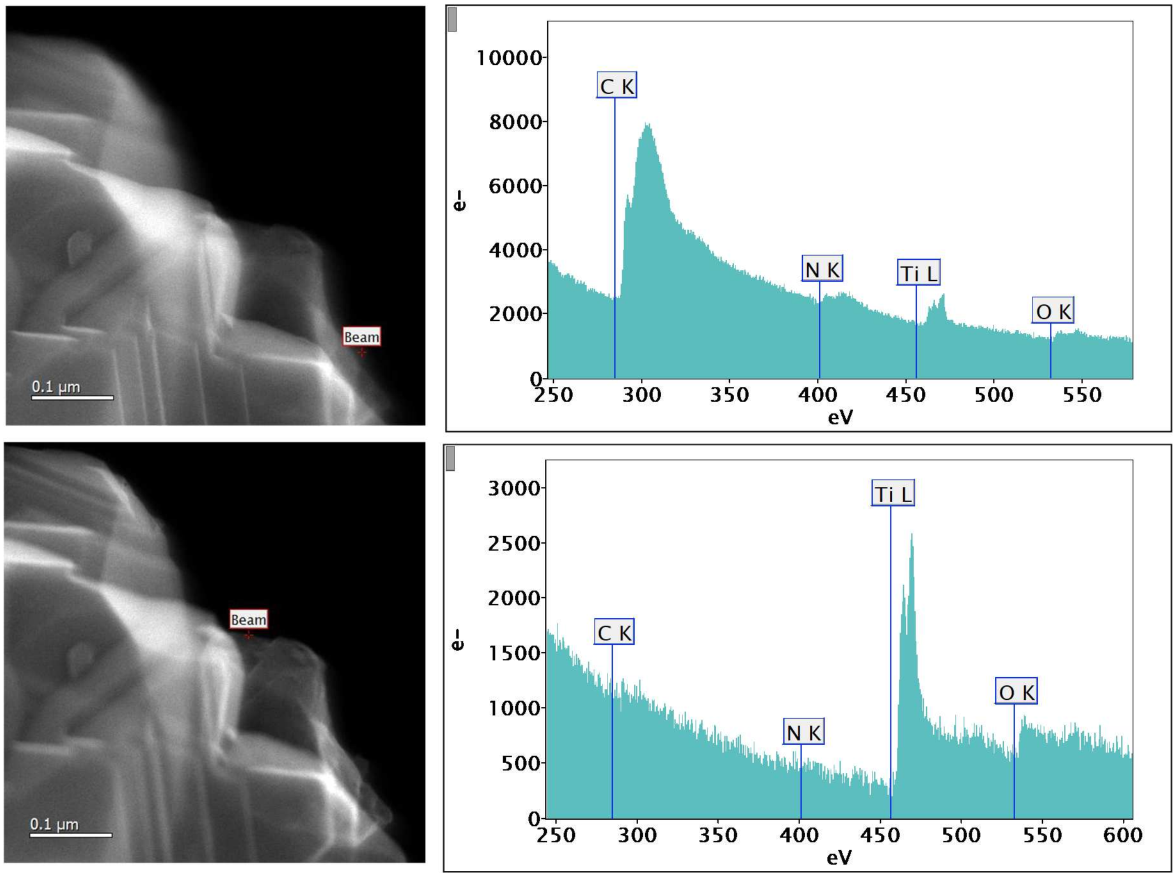


**Figure S2**. EELS analysis of LTO-CC3

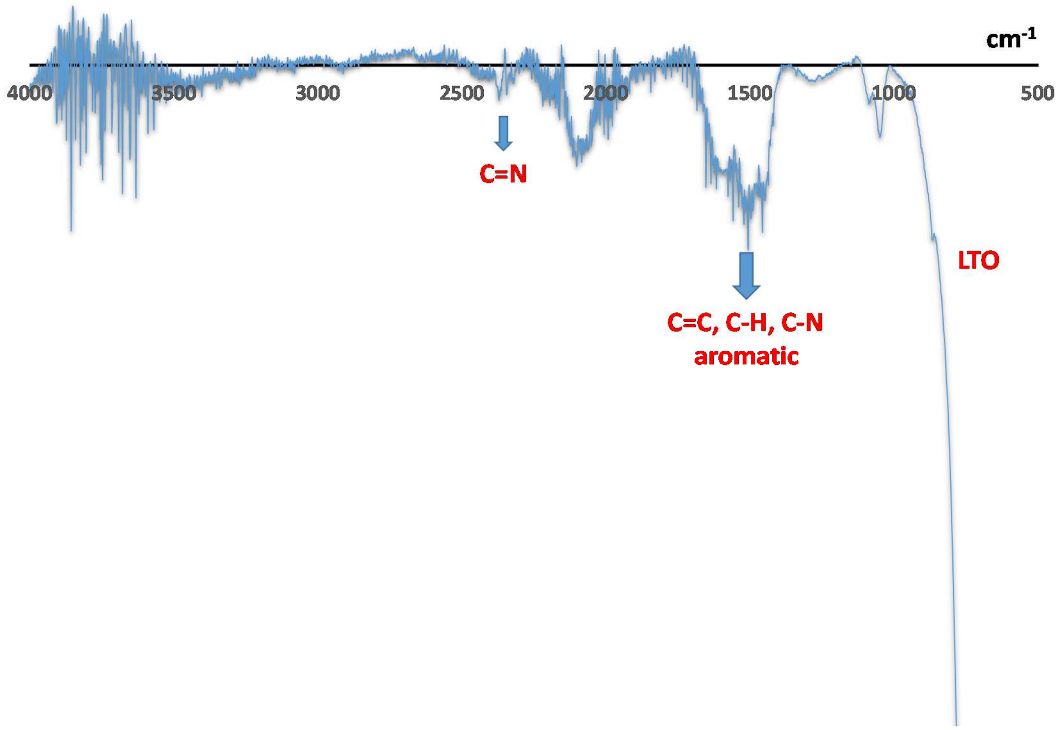


**Figure S3**. FTIR spectrum of LTO-CC3

Ref.: W. Li et al., J. Mater. Chem. A, 2014, 2, 2110

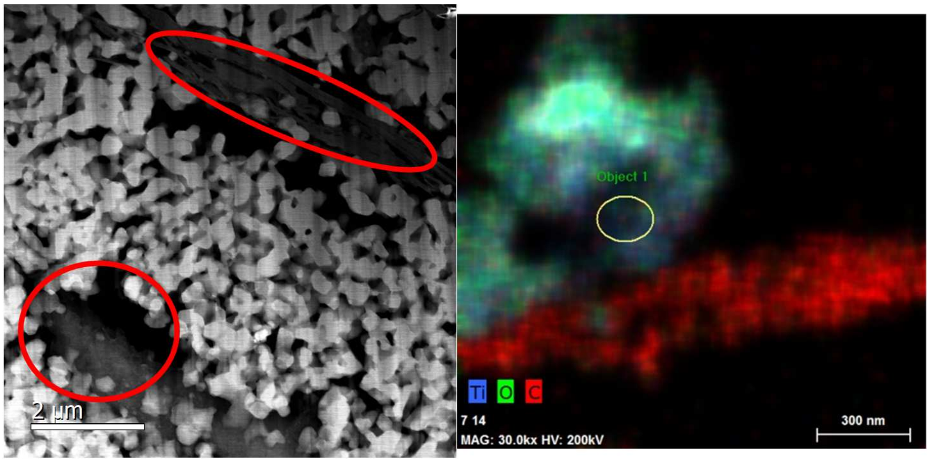


**Figure S4.** Images of LTO-CC reference by TEM. Left: Images of fibbed particles, red circles show carbon chunks. Right: mapping of Ti, O and C.

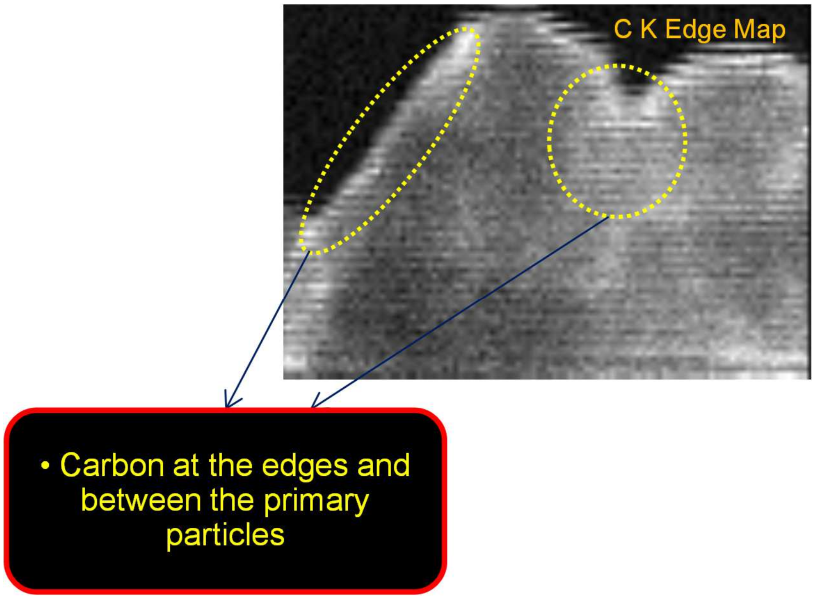


**Figure S5.** Images by HR-TEM of primary particles

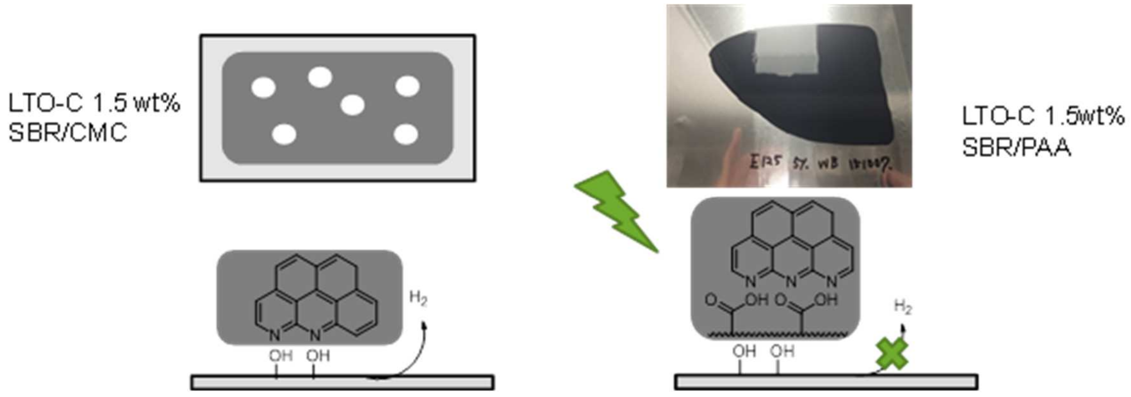


**Figure S6**. Schematic representation of the effect of PAA on N-Doped carbons

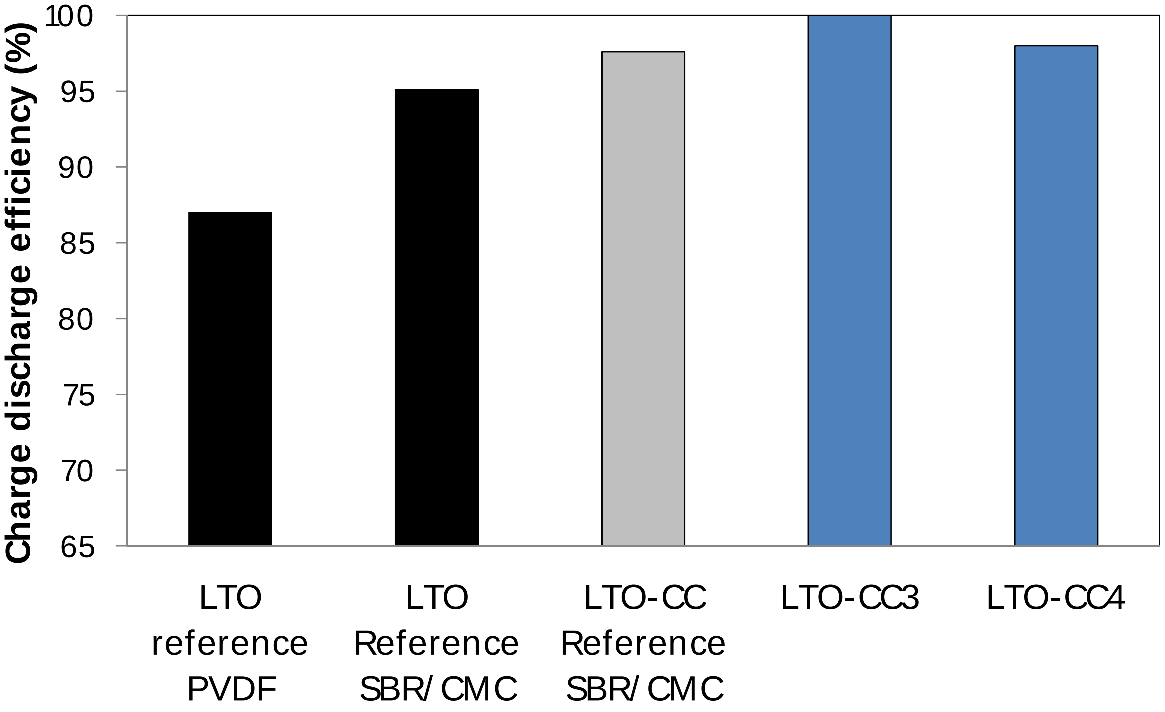


**Figure S7.** Retention capacities after float test at 45°C.
